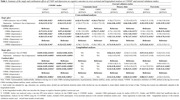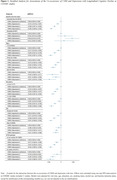# Additive Impact of Cardiometabolic Multimorbidity and Depression on Cognitive Decline: Findings from Multi‐Regional Cohorts and Generalization from Community to Clinic

**DOI:** 10.1002/alz.087254

**Published:** 2025-01-09

**Authors:** Xuhao Zhao, Yifan Yan, Darren M. Lipnicki, Ting Pang, Christopher Chen, Tien Yin Wong, Ching Yu Cheng, Narayanaswamy Venketasubramanian, Eddie Chong, Erico Costa, Richard B. Lipton, Mindy J. Katz, Karen Ritchie, Isabelle Carriere, Nikolaos Scarmeas, Oye Gureje, Hugh C Hendrie, Sujuan Gao, Ricardo Oliveira Guerra, Elena Rolandi, Steffi G. Riedel‐Heller, Mary Ganguli, Allison E Aiello, Roger Chun‐Man Ho, Pascual Sanchez‐Juan, Antonio Lobo, Perminder S. Sachdev, Xiaolin Xu, Xin Xu, Cohort Studies of Memory in an International Consortium (COSMIC)

**Affiliations:** ^1^ School of Public Health, the Second Affiliated Hospital of School of Medicine, Zhejiang University, Hangzhou, Zhejiang China; ^2^ Centre for Healthy Brain Ageing, Discipline of Psychiatry & Mental Health, School of Clinical Medicine, University of New South Wales, Sydney, NSW Australia; ^3^ Memory, Ageing and Cognition Centre, National University Health System, Singapore Singapore; ^4^ National University Health System, NUHS, Singapore, Singapore Singapore; ^5^ Tsinghua Medicine, Tsinghua University, Beijing, Beijing China; ^6^ Singapore Eye Research Institute, Singapore National Eye Centre, Singapore, Singapore Singapore; ^7^ School of Clinical Medicine, Beijing Tsinghua Changgung Hospital, Beijing, Beijing China; ^8^ Raffles Neuroscience Centre, Raffles Hospital, Singapore Singapore; ^9^ Memory, Ageing, and Cognition Centre (MACC), Department of Pharmacology, Yong Loo Lin School of Medicine, National University of Singapore, Singapore, Singapore Singapore; ^10^ Instituto René Rachou da Fundaçaõ Oswaldo Cruz, Rio de Janeiro Brazil; ^11^ Department of Neurology, Albert Einstein College of Medicine, Bronx, NY USA; ^12^ Albert Einstein College of Medicine, Bronx, NY USA; ^13^ Institut for Neurosciences of Montpellier, University Montpellier, National Institute for Health and Medical Research, Montpellier France; ^14^ Institute for Neurosciences of Montpellier, University of Montpellier, INSERM, Montpellier France; ^15^ 1st Department of Neurology, Aiginition Hospital, National and Kapodistrian University of Athens Medical School, Athens Greece; ^16^ Department of Neurology, Columbia University, New York, NY USA; ^17^ WHO Collaborating Centre for Research and Training in Mental Health, Neurosciences and Substance Abuse, Department of Psychiatry, University of Ibadan, Ibadan Nigeria; ^18^ Indiana University School of Medicine, Indianapolis, IN USA; ^19^ Indiana Alzheimer’s Disease Research Center, Indianapolis, IN USA; ^20^ Department of Physical Therapy, Federal University of Rio Grande do Norte, Rio Grande do Norte Brazil; ^21^ Golgi Cenci Foundation, Abbiategrasso Italy; ^22^ University of Pavia, Pavia Italy; ^23^ Institute of Social Medicine, Occupational Health and Public Health (ISAP), Medical Faculty, University of Leipzig, Leipzig, Saxony Germany; ^24^ School of Medicine, University of Pittsburgh, Pittsburgh, PA USA; ^25^ Robert N. Butler Columbia Aging Center, Department of Epidemiology, Mailman School of Public Health, Columbia University, New York, NY USA; ^26^ Department of Psychological Medicine, National University of Singapore, Singapore Singapore; ^27^ Neurology Department, Hospital Universitario Marqués de Valdecilla – IDIVAL – University of Cantabria ‐ CIBERNED, Madrid Spain; ^28^ Department of Medicine and Psychiatry, Universidad de Zaragoza, Zaragoza Spain; ^29^ Instituto de Investigación Sanitaria Aragón, Zaragoza Spain; ^30^ Centre for Healthy Brain Ageing (CHeBA), University of New South Wales, UNSW Sydney, NSW Australia; ^31^ School of Public Health, Faculty of Medicine, The University of Queensland, Brisbane Australia

## Abstract

**Background:**

To estimate the additive associations of cardiometabolic multimorbidity (CMM) and depression on long‐term cognitive trajectory in multi‐regional cohorts and validate the generalizability of the findings in varying clinical settings.

**Method:**

Data harmonization was performed across 14 longitudinal cohort studies within the Cohort Studies of Memory in an International Consortium (COSMIC) group, spanning North America, South America, Europe, Africa, Asia, and Australia. Three external validation studies with distinct settings were employed to assess generalizability. Cross‐sectional and longitudinal analyses were conducted. CMM was defined as: 1) CMM5: ≥ 2 among hypertension, hyperlipidemia, diabetes mellitus, stroke, and heart disease and 2) CMM3 (aligned with previous studies): ≥ 2 among diabetes mellitus, stroke, and heart disease. Depression was identified using the Geriatric Depression Scale, Center for Epidemiological Studies‐Depression scale, or medical history. A one‐step individual participant data meta‐analysis was utilized to investigate associations between the co‐occurrence of CMM and depression and cognitive outcomes in the COSMIC studies. Stratified analyses were conducted based on baseline dementia status, demographics, and APOE genotype. Repeated analyses were performed in external validation studies for generalization.

**Result:**

Of the 32,450 older adults in the 14 COSMIC cohorts, we included 31,243 participants with complete data on CMM, depression, and cognitive assessment for cross‐sectional analyses. Among them, 23,242 who had at least 1 follow‐up cognitive assessment were included in the longitudinal analyses. From the three external studies we included 1964 participants, representing 3 multi‐ethnic Asian elderly cohorts (community cohort, memory clinic cohort, and stroke cohort). In the COSMIC studies analysis, the co‐occurrence of CMM and depression was associated with both cross‐sectional cognitive performance (β = ‐0.20, 95%CI = (‐0.25,‐0.16) for CMM5 and depression, β = ‐0.17, (95%CI = ‐0.044,‐0.031) for CMM3 and depression), and rate of cognitive decline (β = ‐0.038, 95%CI = (‐0.25,‐0.16) for CMM5 and depression, β = ‐0.023, (95%CI = ‐0.036, ‐0.009) for CMM3 and depression). This combined effect remained consistent across different subgroups particularly among participants without dementia. These findings were reproduced in the three external validation studies.

**Conclusion:**

Our study demonstrated an additive effect between CMM and depression on cognitive decline. Targeting both cardiometabolic and psychological conditions could lead to greater effectiveness in delaying or preventing cognitive decline.